# Reviewed article: Souza ASS, Cavalcante JR, Proença R, Rodrigues IA, Frank CHM, Freitas DRC, Filho EBB, Maciel EL, Garcia MHO. History of the implementation of public health emergency management in Brazil. Epidemiol Serv Saude. 2025:34;e20240498

**DOI:** 10.1590/S2237-96222025v34e20240498.b

**Published:** 2025-05-02

**Authors:** Andréia Insabralde de Queiroz-Cardoso

**Affiliations:** UFMS, INISA

**Table:** 

Rating	Excellent	Good	Average	Below average	Poor
Interest		✓			
Quality				✓	
Originality	✓				
Overall				✓	

**Table:** 

Questionnaire	Yes	No	Not applicable
Does the manuscript contain new and significant information to justify publication?	✓		
Does the Abstract (Summary) clearly and accurately describe the content of the article?	✓		
Is the problem significant and concisely stated?	✓		
Are the methods described comprehensively?		✓	
Are the interpretations and conclusions justified by the results?		✓	
Is adequate reference made to other work in the field?	✓		

## Manuscript Structure:

Length of article is: Adequate

Number of tables is: Adequate

Number of figures is: Adequate

Please state any conflict(s) of interest that you have in relation to the review of this paper (state “none” if this is not applicable): None

## Comments

Caro autor, seu manuscrito possuí relevância e originalidade. Para melhorias, sugerimos que revise a fluidez lógica e sequencial das ideias entre parágrafos. As frases precisam ser diretas e sem anunciar o tema do parágrafo. Verifique erros de ortografia e gramática em todo o texto. Assegure que as regras de pontuação e espaçamento foram observadas. Seguem algumas observações:

- Introdução: rever o 3º parágrafo, o qual faz citação de portaria ministerial, visto que pode ser melhor descrito para diminuir similaridade.

- Resultados: rever a escrita do parágrafo com a citação 23, referente ao Plano de respostas às emergências em saúde pública.

- Discussão: 

1. Rever o 2º parágrafo referente ao EpiSUS devido similaridade, citação 9 e 26. 

2. Rever a escrita referente a citação 21 a qual aparece de forma quase sequencial, separada apenas por um parágrafo no qual não consta autoria, mas descreve o trabalho do CIEVS (fazer citação e referência). 

3. Reescrever o 5º parágrafo da página 5 (Relatório iThenticate), pois todo o parágrafo é feito com base em apenas uma citação repetida duas vezes, e também o 6º parágrafo da página 5 que apresenta grande similaridade, citação 32. 

4. Observa-se que em toda discussão existem parágrafos descritos inteiramente sem citações, o que fragiliza o texto, pois o mesmo não elucida evidências de informação científicas, sendo necessário o embasamento e robustez para uma publicação consistente.

5. Sugerimos que no último parágrafo do manuscrito responda de forma clara a pergunta de pesquisa e objetivo para melhor finalização e compreensão da revisão desenvolvida.

Segue o Relatório iThenticate, para adequações e melhorias do manuscrito. 

